# Evaluation of marking of peer marking in oral presentation

**DOI:** 10.1007/s40037-016-0254-8

**Published:** 2016-03-07

**Authors:** Dietmar Steverding, Kevin M. Tyler, Darren W. Sexton

**Affiliations:** Norwich Medical School, University of East Anglia, Norwich Research Park, Norwich, UK; Norwich Medical School, currently: School of Pharmacy and Biomolecular Sciences, Liverpool John Moores University, Liverpool, UK

**Keywords:** Summative assessment, Peer marking, Oral presentation

## Abstract

**Background:**

Peer marking is an important skill for students, helping them to understand the process of learning and assessment. This method is increasingly used in medical education, particularly in formative assessment. However, the use of peer marking in summative assessment is not widely adopted because many teachers are concerned about biased marking by students of their peers.

**Objective:**

The aim of this study was to investigate whether marking of summative peer assessment can improve the reliability of peer marking.

**Methods:**

In a retrospective analysis, the peer-marking results of a summative assessment of oral presentations of two cohorts of students were compared. One group of students was told that their peer marks would be assessed against a benchmark consisting of the average of examiner marks and that these scores together with the peer and examiner marks would form their final exam results. The other group of students were just informed that their final exam results would be determined based on the examiner and peer marks.

**Results:**

Based on examiner marks, both groups of students performed similarly in their summative assessment, agreement between student markers was less consistent and more polar than the examiners. When compared with the examiners, students who were told that their peer marking would be scored were more generous markers (their average peer mark was 2.4 % points higher than the average examiner mark) while students who were not being scored on their marking were rather harsh markers (their average peer mark was 4.2 % points lower than the average examiner mark), with scoring of the top-performing students most affected.

**Conclusions:**

Marking of peer marking had a small effect on the marking conduct of students in summative assessment of oral presentation but possibly indicated a more balanced marking performance.

## Essentials

- Students whose peer marking is assessed mark more generously in summative assessments of oral presentations.
- The effect is greatest for top-performing students who tend to be marked more harshly in summative assessment of oral presentations when peer marking is not subjected to evaluation.
- There is a significant but marginal inflationary effect from assessor moderation of peer marking by students in summative assessments of oral presentations.

## Introduction

Peer assessment is increasingly used in higher medical education as it is an effective method to improve self-directed learning and reflection [1–3]. It usually involves the assessment and evaluation of the work of a fellow student followed by comparison of the work with predefined standards which then allows to identify gaps in knowledge. This method works very well in formative assessments designed to improve students’ independent and reflective learning and to engage students actively in the learning process and in the development of communication, teamwork and presentation skills [4]. In addition, most students are willing to participate in peer assessment and enjoy the process [5].

However, peer marking is less used in summative assessment. This is probably because summative assessment is characterized as evaluation of learning at the end of an instructional unit [6]. As the stakes are often high, teachers have understandable reservations with inclusion of peer marking in summative assessments. In addition, teachers may fear that students would mark their peers too leniently, thus falsely inflating examination grades. But it may also be possible that students may mark their peers more strictly and relative polarity in student peer reviewing has been previously observed [7]. To prevent unfair peer marking, the marks given by the students could be assessed against a benchmark set by the teachers’ marks and included in their final score.

It is obvious that peer marking is not practical for all summative assessments, e.g. written exams. However, there are many other forms of summative assessments in the modern medical curriculum [8], some of which may prove useful. Here we describe the retrospective analysis of peer marking of summative assessments of a teaching unit on presentation skills. In this study two groups of students were compared: the peer marks of the first group were graded and the students were informed about this while the peer marks of the second group remained ungraded. The results show that peer marking in certain summative assessments is feasible and that students generally assess their peers in an unbiased fashion.

## Methods

### Study design

This pilot study retrospectively analyzed the results of summative assessments of the ‘Research Presentation’ part of the postgraduate module ‘Transferable Skills for Research’ taught for medical health professionals at the University of East Anglia. The Research Presentation part consisted of several sessions teaching students about the different methods of presenting research results. At the end of the module the students were assessed on a 10-minute oral presentation accompanied by a PowerPoint slide show. The presentation was marked in two categories: visual aids and presentation skills. The individual marking criteria for the visual aids category included ‘font size’, ‘font style’, ‘amount of text’, ‘figures/tables’, ‘background of slides’, ‘use of colours’ and ‘use of animation’ while those for the presentation skills comprised ‘background information’, ‘presentation of results’, ‘conclusion’, ‘timing’, ‘time per slide’, ‘gestures’ and ‘contact with audience’. The marks for each criterion were: 0 = unacceptable, 1 = unsatisfactory, 2 = satisfactory, 3 = good, 4 = excellent. The presentations were simultaneously marked by two members of staff (examiner marks) and by the students (peer marks) taking the module. The examination results of two cohorts of students from two different years were analyzed. The students of one year (group M) were informed that their peer marks would be scored against the mean examiner marks as benchmark. The students were also briefed that the weighting of their final marks for their presentation would consist of 60 % examiner marks, 25 % peer marks and 15 % marks for peer marking. The students of another year (group U) were just informed that they would carry out peer marking and that the peer marks would be included in their final mark for their presentation at a weighting of 75 % examiner marks and 25 % peer marks. The cohorts of the two years were chosen because both cohorts consisted of nine students. Statistical analyses were performed to see whether the marking of the peer marks influenced the scoring attitude of students by comparing the peer-marking results of the cohorts with each other and with the examiner marks.

### Ethics

This study was approved by the Faculty of Medicine and Health Sciences Research Ethics Committee of the University of East Anglia (reference: 20142015 69 SE).

## Results

**Fig. 1:**
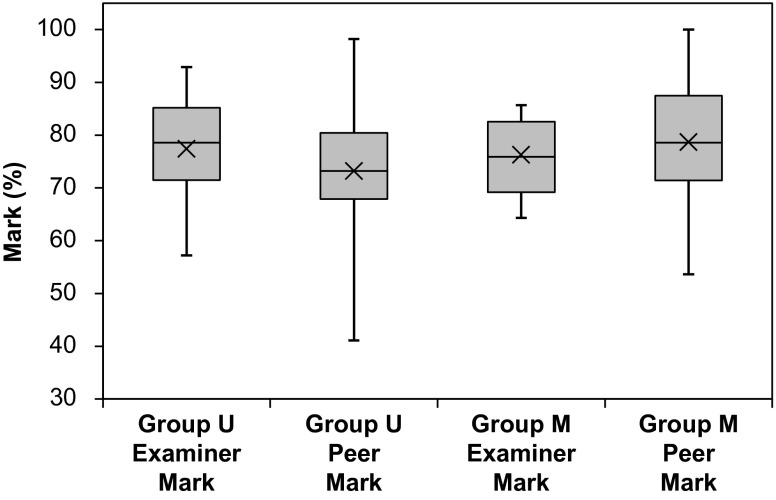
Box-and-whisker plot of examiner and peer marks for group U (peer marks were ungraded) and group M (peer marks were graded). The plot shows the median (inside bar), the mean (cross), the first and the third quartiles (bottom and top of the box), and the minimum and maximum (end of whiskers)

In order to be able to determine whether scoring of peer marks would affect the peer marking of students, an essential prerequisite was that the two student groups U and M were matchable with respect to their exam performance. Both groups were similarly marked by the examiners indicating that their exam performances were comparable (Fig. 1). The mean examiner marks were 77.4 ± 10.6 % (95 % CI 72.4–82.4 %) for the group U students and 76.3 ± 7.3 % (95 % CI 71.4–81.2 %) for the group M students. The observed difference of 1.1 percentage points between the mean examiner marks of the two groups was statistically insignificant (*p* = 0.263; Student’s t-test). Compared with the respective mean examiner marks, the mean peer marks of the group U students (73.2 ± 10.7; 95 % CI 70.7–75.8 %) was 4.2 % points lower, while the mean peer marks of the group M students (78.7 ± 11.1 %; 95 % CI 72.2–81.2 %) was 2.4 % points higher (Fig. 1). These differences, however, were statistically not significant (group U, *p* = 0.14; group M, *p* = 0.38; Student’s t-test). Another observable difference was that the min/max range for the student groups was much bigger than that for the examiners (Fig. 1). In addition, when the mean peer marks of group U and M were compared with each other, the observed difference between the marks was highly significant (*p* = 0.0035; Student’s t-test).

**Fig. 2:**
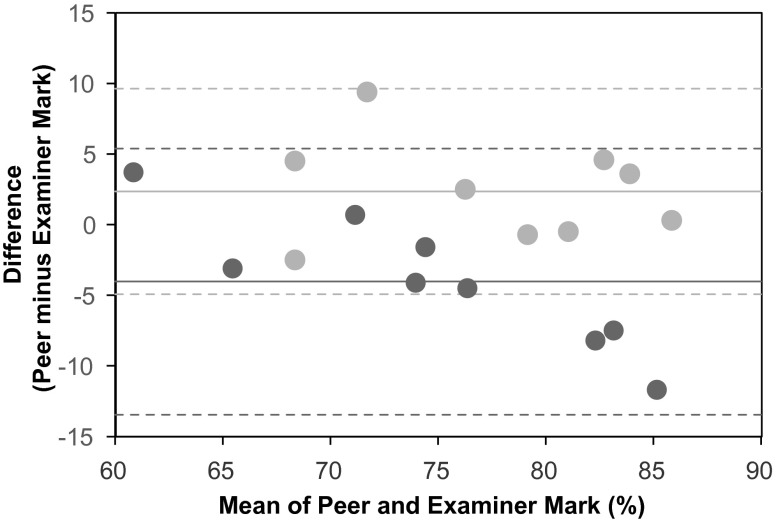
Bland-Altman plot of peer minus examiner marks against mean of peer and examiner marks for group U students (*dark grey*) and group M students (*light grey*). Solid lines, means; dashed lines, means ± SD

Next, peer and examiner marks were analyzed with a Bland-Altman plot [9]. This method plots the differences between two measures against the average of the two measures and determines whether there is any systematic bias (i.e., in our case whether there is a tendency for the student marking to be lower or higher than the examiner marking). The Bland-Altman analysis showed that the peer and examiner marks of both groups spread differently (Fig. 2). The different mean marks in groups U and M were − 4.03 ± 4.71 (95 % CI − 7.01 to − 1.06) and + 2.36 ± 3.64 (95 % CI − 0.62–5.33) percentage points, respectively. The discrepancy in these mean marks was statistically significant (*p* = 0.005; Student’s t-test).

**Fig. 3:**
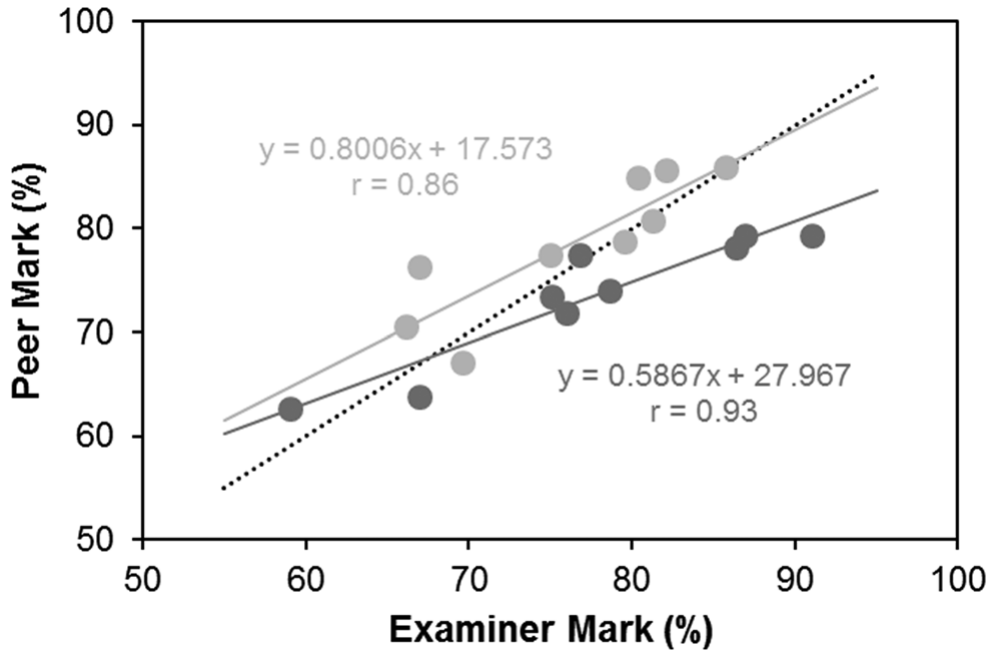
Scatterplot of examiner and student marks for group U students (*dark grey*) and group M students (*light grey*). Dotted line, line of equality (if there were total agreement between marks); solid lines, regression line. Regression function and correlation coefficient for the mark of both groups are shown

Linear regression analysis showed that there was generally a good correlation between the peer and examiner marks (> 0.85; Fig. 3). However, the regression line of both groups deviated from the line of equality (Fig. 3). In the case of the group U students the deviation indicates that they marked their better peers more harshly than the examiners. For group M students there was a trend that they marked their weaker peers more kindly than the examiners.

## Discussion

This retrospective pilot study has shown that peer marking is feasible in summative assessments. An attempt was made to improve the reliability of peer marking by introducing marks for the peer-marking quality. Interestingly, our study indicates that students whose peer marking was not assessed (group U) were more likely to mark their peers harshly than those students who were told that their peer marking would be assessed (group M) and a mark for the quality of their peer marking would be included in their final score. This was somewhat unexpected as we had assumed that group U students would mark their peers more generously than group M students. This is because students can be influenced by concerns over recognizing the work of peers and subsequently award biased higher scores. This phenomenon is known as ‘friendly marking’ [10]. This was recently shown in a study evaluating the peer marking of laboratory reports of first-year undergraduates, where peer-awarded marks exceeded staff marks by an average of 2.5–3.0 percentage points [11]. However, the contrary that students mark harsher than examiners has also been reported [2]. The problem of ‘friendly marking’ may be overcome by anonymizing the work to be marked. However, this was not possible in our study as oral presentations were assessed.

Given that there was reasonable reliability between examiner and peer marks of both student groups, the introduction of assessing peer marks actually affected the marking attitude of students only marginally. Only group U students who performed very well in the exam were affected in the way that they were marked down by their peers. As the weighting of peer marks was 25 % for group U students, these top students lost an average of 1.9–2.9 percentage points on their final exam result which would not have impacted on their overall final grade. However, by reducing the weighting of peer marks, the impact on final exam results can be limited. In a recent study 70 % of students agreed that peer assessment would be acceptable if it contributes only a little (up to 5 %) to the overall final mark [11].

Previous studies have shown that students find it challenging to mark their peers and express concerns about their peers’ lack of expertise which might result in lost marks [2, 12]. It was suggested early on that the process of peer marking requires training of students in specialized skills of summarizing and evaluation [13]. In our study, the students were taught beforehand about what to consider for a good presentation. Emphasis was placed on visual aids, presentation skills and the assessing criteria for their summatively assessed presentation. In addition, before the students gave their presentations, a formative assessment of a related presentation with verbal feedback was carried out. Although the students did not see the marking sheet until the examination day, it seemed that the students did not have any problems with it as no clarifying questions regarding the marking sheet were asked. Our students may also have been quite confident about peer marking as they were postgraduates who may have experienced some kind of peer marking previously during their undergraduate studies.

It has been reported that students find peer marking generally positive, especially in formative assessments [2, 11, 14]. The students commented that peer marking helped them in increasing their understanding of the subject matter and of the examination techniques required [2, 11]. Whether our students regarded the peer-assessment process positively remains unanswered because no questioning of the students was ever intended as this was a retrospective analysis. For future studies it would be interesting if students were to also provide narrative reports to justify their scores. This would make them think about why they have awarded a certain score and thus help them to understand the evaluation process. Indeed, it may further modify their scores if justifications were required.

One limitation of our study is the very small sample size. Although the results were statistically significant, given that the observed effect was small, it is quite possible that the findings were just a result of selection bias, i.e. the findings represented just the differences between the two study groups rather than the effect of marking of peer marking. However, as this was not a predesigned study and therefore the students did not know that their peer assessment would ever be evaluated, the obtained findings may be robust. In a prospective study, the knowledge that their peer marking will be evaluated might have introduced bias in the student marking (Hawthorne effect).

When trying to successfully implement peer marking in educational settings some general practical recommendations should be followed [15]. Students’ prior experience with peer assessment must be considered. For example, first-year students are less likely to have experienced peer marking compared with postgraduates. The students should be informed before the start of the course that peer marking is part of the assessment process. This can be highlighted in the course’s description and/or explained during the first session. Formative assessments should be incorporated to familiarize students with the assessment process and performance criteria. Peer marking should be performed in an anonymized way. Concerns regarding bias and unfair marking should be discussed and measures to prevent this should be provided. For example that peer marks are assessed as outlined in this study and that peer marking contributes only part of the final mark.

## Conclusions

This pilot retrospective study has shown that marking of summative peer assessment only has a small effect on marking performance. In particular, top students benefited from the introduction of assessment of peer marking. One conclusion from this study is that further investigations are required to confirm this retrospective study, which has the possibility of small sample size error.
